# Synthesis and Antibacterial Activity of Triphenyltinbenzoate

**DOI:** 10.4103/0250-474X.73923

**Published:** 2010

**Authors:** M. K. Choudhury, B. Zewdie

**Affiliations:** Department of Pharmaceutical Chemistry, School of Pharmacy, Addis Ababa University, Addis Ababa, PO Box 1176, Ethiopia

**Keywords:** Antibacterial activity, MIC, synthesis, triphenyltinbenzoate, zone of inhibition

## Abstract

Triphenyltinbenzoate was synthesized using triphenyltinchloride and silver benzoate prepared from sodium benzoate. The structure of the synthetic compound was elucidated by spectral and C, H analysis. The antibacterial activities of the organotin compound were determined against four bacteria namely *Escherichia coli* (ATCC 25922), *Staphylococcus aureus* (ATCC 25923), *Streptococcus pyogenes* (clinical isolate) and *Pseudomonas aeruginosa* (ATCC 27853) in vitro experiment. All the bacteria were inhibited at a concentration of 200 μg/ml and 20 μg/ml in dimethylsulphoxide solution and the minimum inhibitory concentration was found to be same, 7.5 μg/ml for *Escherichia coli*, *Staphylococcus aureus*, *Streptococcus pyogenes* and 10 μg/ml for *Pseudomonas aeruginosa*.

Organotin compounds show a large spectrum of biological activities. It was observed that several organotin compounds were found to be antineoplastic and antiviral agents, used commercially as bactericides, fungicides and acaricides[1,3]. Some were potential biocides[4], they exhibited significant fungicidal activities[5], triphenyltin acetate has been commercially marketed as a fungicide[6]. The antifungal activities of organotin compounds were also reported[7,8]. Tributyltin compounds were first used as mollusicides to kill several species of fresh snails that were intermediate hosts of the parasitic worm schistoma which transmits the disease schistomiasis to human[9]. The antibacterial and antifungal activities of trimethyltinbenzoate-4-picoline were reported earlier[10,11]. Triphenyltin acetate and triphenyltin hydroxide are used as agricultural fungicides[12].

Triphenyltin chloride obtained from Merck-Schuchardt, Germany was used for the synthesis and other chemicals were procured from BDH, England. Mueller-Hinton Agar and Mueller Hinton Broth were used for antibacterial activities. IR spectra were recorded in Perkin Elmer spectrum BX spectrophotometer using KBr discs. ^1^H and ^13^C-NMR were measured in Bruker Advance DMX 400 FT-NMR using tetramethylsilane (TMS) as an internal standard and CDCl_3_ as a solvent.

Silver benzoate was prepared by the addition of an aqueous solution silver nitrate (2.89 g, 17 mmol, 10 ml) to an aqueous solution of sodium benzoate (2.4 g, 17 mmol, 10 ml) and the mixture was stirred at room temperature for 2 h in the dark. The white precipitate formed was filtered, dried, gave 3.25 g (85% yield).

Triphenyltinbenzoate (TPTB, fig. 1) was sythesized by the reaction between silver benzoate (2.4 g, 10 mmol) and triphenyltin chloride (4.04 g, 10 mmol) in chloroform (150 ml). The mixture was stirred for 48 h in a conical flask covered with an aluminium foil in the dark and the precipitate (AgCl) formed was filtered. The clear filtrate was evaporated to give a solid, crystallized from chloroform, 4.44 g (90% yield), mp 79-80°. The R_f_ values of triphenyltinbenzoate and triphenyltinchloride were 0.48 and 0.40 respectively in TLC (benzene-chloroform-methanol, 10:9:0.5).

**Fig. 1:**
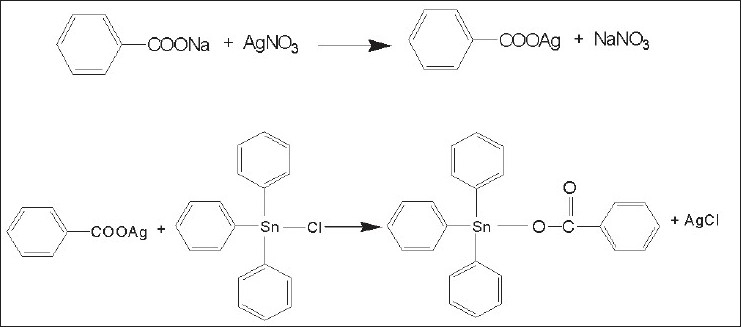
Synthesis of triphenyltinbenzoate

IRv_max_ (KBr, cm^-1^): 1429 and 1626 (strong benzoate bands), 1126 (Sn-Ph deformation bands), 996 (Sn-O-C band)[13]. ^1^H-NMR (δ, CDCl_3_): 7.38 (m, 4H, ortho and meta-H of O-CO-Ph), 7.47 (m, 9H, meta and para-H of Sn-Ph), 7.82 (m, 6H, ortho-H of Sn-Ph), 8.15 (m, 1H, para-H of O-CO-Ph). ^13^C-NMR (δ,CDCl_3,_): 128.25 (meta-C of O-CO-Ph), 130.26 (meta-C of Sn-Ph), 130.69 (ortho-C of O-CO-Ph), 130.73 (para-C of Sn-Ph), 137.02 (ortho-C of Sn-Ph), 132.76 (para-C of O-CO-Ph), 138 (4QC of Sn-Ph and O-CO-Ph), 173 (CO of O-CO-Ph). In DEPT ^13^C-NMR the QC (138) and CO group (173) disappeared. Analysis: C_25_H_20_O_2_Sn required: C, 63.73%; H, 4.28%; Sn, 25.19%. Found: C, 63.63%; H, 4.34%; Sn, 25.11%.

The antibacterial activity was determined against *Escherichia coli* (ATCC 25922), *Staphylococcus aureus* (ATCC 25923), *Streptococcus pyogenes* (clinical isolate) and *Pseudomonas aeruginosa* (ATCC 27853) using the agar diffusion method. Mueller-Hinton Agar (MHA) medium was prepared according to manufacturer’s instruction. Solutions of three different concentrations of the TPTB compound, 200 μg/ml, 20 μg/ml and 2 μg/ml were prepared using 100% DMSO as solvent. Five Petri dishes (120 mm diameter) were used for the experiment. MHA medium (20 ml) was poured into each Petri dish and then sterilized in autoclave at 121° for 15 min. They were cooled and then each plate was inoculated with different bacteria culture. Five wells were dug on each inoculated plate using a sterilized cork borer (5 mm internal diameter). Two wells in each plate were filled with 0.5 ml test solution of triphenyltinbenzoate having concentrations 200 μg/ml and 20 μg/ml, the third and fourth wells filled with ciprofloxacin (standard antibacterial drug) having concentrations 4 μg/ml, 2 μg/ml and the fifth well with DMSO (100%, as a control) for the sake of comparison. The plates were then incubated at 37° for 24 h. After incubation the cultures were examined for the evidence of inhibition that appeared as clear zones of varying diameters according to concentration around the wells and measured in mm.

The minimum inhibitory concentration (MIC) was determined using tube dilution method. Serial dilution of test compound, triphenyltinbenzoate and ciprofloxacin were prepared in test tubes containing nutrient Mueller-Hinton broth as diluent. The lowest concentration that showed inhibition of growth of each organism was further diluted to different lower concentrations in order to determine the MIC. Each bacterial isolate was inoculated into different tube accordingly containing the diluted compound and then incubated at 37° for 24 h. The tubes were then examined for the presence of growth considering turbidity as criteria. The lowest concentration in each series that showed no growth (no turbidity) of test organism was considered to be the MIC of that organism.

At 200 μg/ml and 20 μg/ml concentrations of triphenyltinbenzoate, the zone of inhibition for *Escherichia coli* were 25.5 mm and 20 mm, for *Staphylococcus aureus* were 24 mm and 19.5 mm, for *Streptococcus pyogenes* were 19.5 mm and 14.5 mm, for *Pseudomonas aeruginosa* were 11.5 mm and 9.5 mm respectively. At 4 μg/ml and 2 μg/ml concentrations of ciprofloxacin, the zone of inhibition for *Escherichia coli* were 30.4 mm and 28.3 mm, for *Staphylococcus aureus* were 30.5 mm and 28.1 mm, for *Streptococcus pyogenes* were 26.6 mm and 24.5 mm, for *Pseudomonas aeruginosa* were 34.5 mm and 32.3 mm respectively (Table 1). DMSO (100%) and concentrations of TPTB at 2 μg/ml and 0.2 μg/ml did not show any zone of inhibition for any bacteria.

**TABLE 1:** ZONE OF INHIBITION AND MIC VALUES OF TPTB AND CIPROFLOXACIN AGAINST DIFFERENT BACTERIA

Compound	TPTB (mm)			Ciprofloxacin (mm)		
	200 μg/ml	20 μg/ml	MIC (μg/ml)	4 μg/ml	2 μg/ml	MIC (μg/ml)
*Escherichia coli*	25.5	20	7.5	30.4	28.3	0.0156
*Staphylococcus aureus*	24	19.5	7.5	30.5	28.1	0.25
*Streptococcus pyogenes*	19.5	14.5	7.5	26.6	24.5	2
*Pseudomonas aeruginosa*	11.5	9.5	10	34.5	32.3	0.0156

The minimum inhibitory concentration (MIC) of triphenyltinbenzoate for *Escherichia coli*, *Staphylococcus aureus*, *Streptococcus pyogenes*, *Pseudomonas aeruginosa* were found to be 7.5, 7.5, 7.5 and 10 μg/ml, respectively. The MICs of ciprofloxacin for *Escherichia coli*, *Staphylococcus aureus*, *Streptococcus pyogenes*, *Pseudomonas aeruginosa* were found to be 0.0156, 0.25, 2 and 0.0156 μg/ml, respectively (Table 1).

At 200 and 20 μg/ml concentrations of ciprofloxacin, the zone of inhibitions were too large to measure that went out of the scale in the Petri dish used. Therefore, a solution of much lower concentrations of 4 and 2 μg/ml were chosen to overcome the difficulties. From the above results obtained in the experiment it is very clear that the synthetic organotin compound (TPTB) is almost 100 times less active than the standard drug ciprofloxacin. However, the synthetic compound may be useful for some other purposes where a mild active drug is required. The advantage of using organotin compounds is that they are biodegradable that ultimately result in the formation of a non-toxic SnO_2_ (tin dioxide) compound when used for agricultural purposes.
